# *Klebsiella pneumoniae* exhibiting a phenotypic hyper-splitting phenomenon including the formation of small colony variants

**DOI:** 10.3389/fcimb.2024.1372704

**Published:** 2024-03-27

**Authors:** Eyüp Doğan, Katharina Sydow, Stefan E. Heiden, Elias Eger, Georgi Wassilew, Richard A. Proctor, Jürgen A. Bohnert, Evgeny A. Idelevich, Katharina Schaufler, Karsten Becker

**Affiliations:** ^1^ Friedrich Loeffler-Institute of Medical Microbiology, University Medicine Greifswald, Greifswald, Germany; ^2^ Department of Epidemiology and Ecology of Antimicrobial Resistance, Helmholtz Institute for One Health, Helmholtz Centre for Infection Research Helmholtz Center for Infection Research (HZI), Greifswald, Germany; ^3^ Center for Orthopedics, Trauma Surgery and Rehabilitation Medicine, University Medicine Greifswald, Greifswald, Germany; ^4^ Departments of Medical Microbiology/Immunology and Medicine, University of Wisconsin School of Medicine and Public Health, Madison, WI, United States; ^5^ Institute of Medical Microbiology, University Hospital Münster, Münster, Germany; ^6^ University Medicine Greifswald, Greifswald, Germany

**Keywords:** *Klebsiella pneumoniae*, hyper-splitting, small colony variant, hypervirulence, multidrug resistance, in-host evolution, convergence

## Abstract

In this study, we characterized a *Klebsiella pneumoniae* strain in a patient with shrapnel hip injury, which resulted in multiple phenotypic changes, including the formation of a small colony variant (SCV) phenotype. Although already described since the 1960s, there is little knowledge about SCV phenotypes in *Enterobacteriaceae*. The formation of SCVs has been recognized as a bacterial strategy to evade host immune responses and compromise the efficacy of antimicrobial therapies, leading to persistent and recurrent courses of infections. In this case, 14 isolates with different resisto- and morpho-types were distinguished from the patient’s urine and tissue samples. Whole genome sequencing revealed that all isolates were clonally identical belonging to the *K. pneumoniae* high-risk sequence type 147. Subculturing the SCV colonies consistently resulted in the reappearance of the initial SCV phenotype and three stable normal-sized phenotypes with distinct morphological characteristics. Additionally, an increase in resistance was observed over time in isolates that shared the same colony appearance. Our findings highlight the complexity of bacterial behavior by revealing a case of phenotypic “hyper-splitting” in a *K. pneumoniae* SCV and its potential clinical significance.

## 1 Introduction

*Klebsiella pneumoniae*, an opportunistic pathogen known for its ability to cause a wide range of nosocomial and community-acquired infections, has emerged as a significant public health threat due to its strain-specific, extensive arsenal of resistance and virulence factors (Wyres et al., 2020; Antimicrobial Resistance Collaborators, 2022). Infections caused by multi-, extensively-, and pandrug-resistant strains result in high mortality due to limited response to antibiotic therapy, which poses an increasing threat (Ventola, 2015; Navon-Venezia et al., 2017; Avgoulea et al., 2018). Apart from classic strains, a hypervirulent *K. pneumoniae* (hvKp) pathotype occurs and is characterized by invasive, often life-threatening and multiple site infection, characteristically in healthy patients from the general population (Russo and Marr, 2019). In addition, convergent types that successfully combine resistance and hypervirulence represent a “perfect storm” and have been increasingly reported in recent years (Heiden et al., 2020; Lan et al., 2021; Eger et al., 2022).

Beyond typical resistance mechanisms against various antimicrobials, functional resistance mechanisms have been elucidated that lead to antimicrobial treatment failure and foster the development of relapses and persistent infections (Ster et al., 2017). The formation of a biofilm matrix represents one of these mechanisms that facilitates antibiotic tolerance and the generation of bacterial persister cells (Ster et al., 2017). Interestingly, it has been demonstrated that a decrease in capsule biosynthesis, which is crucial for hypervirulent phenotypes, leads to increase *in vitro* biofilm formation and intracellular persistence (Ernst et al., 2020). Another non-classical mechanism leading to antibiotic tolerance is the formation of the small colony variant (SCV) phenotype. SCVs are subpopulations of bacteria that exhibit slow growth, reduced colony size, and altered phenotypic properties compared to their normal-growing counterparts, making them difficult to detect and treat effectively (Proctor et al., 2006; Becker, 2023). Their ability to evade the host’s immune surveillance and to undermine the effectiveness of antimicrobial interventions by host cell internalization results in intracellular persistence, which contributes significantly to the recurrence and chronicity of the infection (Tuchscherr et al., 2011; Kahl et al., 2016). Intracellular persistence have been shown for different human and animal cell types including endothelial and epithelial cells such as keratinocytes and osteoblasts (von Eiff et al., 2001; Strobel et al., 2016). Another pivotal attribute facilitating this phenomenon is their capability to modulate metabolic processes and virulence characteristics (Kriegeskorte et al., 2014; Proctor et al., 2014). Hypermutator SCVs characterized by higher mutation frequencies than wild-type strains and isolated especially from cystic fibrosis (CF) patients (Oliver et al., 2000; Prunier et al., 2003) have also been associated with antibiotic resistance (Schaaff et al., 2003; Besier et al., 2008) and biofilm formation (Morelli et al., 2015).

To date, research has focused on staphylococcal SCVs, while SCVs of Gram-negative bacteria have been investigated in only a few studies and case reports (Proctor et al., 2006). Although the formation of small colonies in *K. pneumoniae* has been noticed during resistance studies against cephalosporins in the mid-1960s (Benner et al., 1965), this issue has not received sufficient attention and detailed research has not been conducted on this subject. The first clearly defined SCV of *K. pneumoniae* (SCV-Kp) in literature was obtained by *in vitro* exposure to gentamicin (Musher et al., 1979). SCV-Kp were also isolated from a patient treated with aminoglycoside antibiotics (Murray and Moellering, 1982). Smaller and non-mucoid colonies were obtained as a result of conjugation-induced mutation in the outer membrane protein of a hypervirulent *K. pneumoniae* isolate (Srinivasan et al., 2012). Another study showed that biofilm-forming *K. pneumoniae* developed heteroresistance to colistin by presenting slow-growing SCV-Kp (Silva et al., 2016).

Here, we report on *K. pneumoniae* isolates displaying 14 different resisto- and morpho-types obtained from an immunocompetent male patient, who had sustained a traumatic injury caused by shrapnel shell fragments. The isolates comprise an initial, mostly susceptible *K. pneumoniae* isolate with typical morphological characteristics isolated from the patient’s urinary specimen. From the urine and tissue samples, 13 additional phenotypes with different combinations of resistance and morphological characteristics including *K. pneumoniae* SCV phenotypes were isolated.

## 2 Materials and methods

### 2.1 Patient data

Sufficient information could not be obtained regarding the period from the patient’s first acetabular and femoral head shrapnel-caused war injury in Ukraine in March 2022, where he underwent hip prosthesis at an external center before his transfer to our orthopedic service in July 2022. Fracture-related joint infection treatment in our hospital continued through November 2022. The administration of antibiotics during this period included piperacillin/tazobactam from July to October, 2022, trimethoprim/sulfamethoxazole from July to August, 2022, cefiderocol from August to November, 2022, and colistin from October to November, 2022. Daptomycin was introduced into the treatment protocol starting from October 2022 upon detection of *Staphylococcus epidermidis* from intraoperatively obtained hip tissue samples and central venous catheter tip, and continued until the patient’s discharge. No other bacteria were isolated from clinical samples during this period. Subsequently, a planned course of post-discharge antibiotic suppression therapy with doxycycline for three months was initiated. The first identification of carbapenem-resistant *K. pneumoniae* (CRKP) occurred in July 2022, followed by the initial detection of SCV-Kp in September 2022. Therefore, we decided to aggregate and systematically assess the entirety of *K. pneumoniae* strains isolated from the patient.

### 2.2 Strain identification

The urine sample obtained from the patient was quantitatively inoculated onto a Columbia agar plate with 5% sheep blood (BD Diagnostics, Heidelberg, Germany) and a MacConkey II-Agar plate (BD Diagnostics) using a 10 µl disposable sterile loop. The plates were then incubated for 48 hours. Tissue samples collected during surgery were inoculated onto Columbia agar plates with 5% sheep blood, MacConkey II-Agar plates, and Mueller Hinton Chocolate agar plates (all from BD Diagnostics). These plates were incubated under capnophilic conditions for up to seven days. The remaining tissue material was inoculated onto Schaedler agar and into BBL Fluid Thioglycollate media (both from BD Diagnostics) and incubated for up to 14 days under anaerobic and capnophilic conditions, respectively.

Preliminary characterization of each phenotype was grounded in colony morphology and minimal inhibitory concentration (MIC) results for antibiotics encompassed within the VITEK® 2 AST card specific to *Enterobacterales* (bioMérieux SA, Marcy l’Étoile, France) according to EUCAST criteria. All *K. pneumoniae* strains, isolated from various patient’s specimens during the period from July to December 2022, were identified by matrix-assisted laser desorption/ionization time-of-flight mass spectrometry (MALDI-TOF MS) utilizing the MALDI Biotyper® sirius system (Bruker Daltonics, Bremen, Germany) with MBT Biotargets 96 (Bruker Daltonics). The presence of carbapenemase-encoding genes was verified by a loop-mediated isothermal amplification (LAMP)-based assay (eazyplex®, AmplexDiagnostics, Gars-Bahnhof, Germany).

### 2.3 Characterization of the phenotypes

@Sequential subcultures of all phenotypic variants were carried out on various agar plates (including Columbia agar + 5% sheep blood, MacConkey agar from BD, and CHROMID® CPS® Elite agar from bioMérieux) to observe whether changes in colony morphology occurred and SCVs remained stable, followed by meticulous analysis of generated phenotypic profiles.

In order to determine colony sizes, each phenotype was inoculated onto 5% sheep blood agar plates in triplicate on different days. After overnight incubation at 35 ± 1°C in ambient air, the diameters of ten colonies of each phenotype were measured and mean values were determined. Additionally, colony morphology in different phenotypes was assessed using the stereo zoom microscope Axio Zoom.V16, equipped with the objective Plan Z 1.0x/0.25 and the Axiocam 305 camera (Zeiss, Oberkochen, Germany). After Gram staining, single cells from different phenotypes were observed in transmission light by the Axio Imager.Z2m microscope with the oil immersion objective Plan-APOCHROMAT 100x/1.4 and Axiocam 305 camera (Zeiss).

### 2.4 Antimicrobial susceptibility testing

In addition to the initial VITEK® 2 AST, the MICs of a standardized set of antibiotics (**Table 1**) were determined by the broth microdilution (BMD) method using cation-adjusted Mueller–Hinton broth (CAMHB; Micronaut-S 96-well microtiter plates, Merlin, Bornheim-Hersel, Germany), and for cefiderocol using iron-depleted CAMHB (UMIC®, Merlin, Bornheim-Hersel, Germany), as recommended by ISO 20776-1, the European Committee on Antimicrobial Susceptibility Testing (EUCAST), and the Clinical and Laboratory Standards Institute (CLSI) guidelines (CLSI, 2018; Standardization, 2019; EUCAST, 2023b). The results were observed following 18 ± 2 hours of incubation at 35 ± 1°C in ambient air. All tests were conducted in triplicate on different days, and median MIC values were computed for analysis. *Escherichia coli* ATCC 25922, *E. coli* ATCC 35218, *K. pneumoniae* ATCC 700603, and *Pseudomonas aeruginosa* ATCC 27853 were used as quality control (QC) strains, and their results were within the QC range throughout the study. EUCAST Clinical Breakpoint Tables v. 13.1 were used for MIC interpretation (EUCAST, 2023a).

**Table 1:** Colony morphology and antimicrobial susceptibility characteristics of the 14 phenotypes of the Klebsiella pneumoniae strain.

14 phenotypes of the *K. pneumoniae* strain					Median minimum inhibitory concentrations (MICs), µg/mL*^a,b^*																		Presence of *bla* genes
Isolate number	Specimen	Date	Colony morphology		PIP	TZP	TEM	FDC	CTX	CAZ	CZA	C/T	IPM	MEM	CIP	LVX	AMK	TGC	CHL	CST	FOF	SXT	
			Color	Surface																			
1-A	Urine	Jul 22	Whitish	Glistening, smooth	≤8	≤4	≤32	≤0.03	≤1	≤1	≤1	≤1	≤1	≤0.125	>2	>2	≤4	0.5	16	≤1	>128	≤1	*bla*_SHV-11_
1-B			Grey	Glistening, smooth	≤8	≤4	≤32	≤0.03	≤1	≤1	≤1	≤1	≤1	≤0.125	>2	>2	≤4	0.5	16	≤1	>128	≤1	
2-A	Intra-operatively obtained hip tissue	Jul 22	Whitish	Glistening, smooth	>16	>64	>128	1	>2	>128	>16	>8	>8	128	>2	>2	8	0.5	>16	≤1	>128	≤1	*bla*_OXA-1,_ *bla*_OXA-9,_ *bla*_OXA-48,_ *bla*_TEM-1D,_ *bla*_CTX-M-15,_ *bla*_NDM-1,_ *bla*_SHV-11_
2-B			Grey	Glistening, smooth	>16	8	≤32	0.06	≤1	≤1	≤1	≤1	≤1	≤0.125	>2	>2	8	≤0.25	≤8	≤1	>128	≤1	*bla*_OXA-9,_ *bla*_TEM-1D,_ *bla*_SHV-11_
3-A	Intra-operatively obtained hip tissue	Aug 22	Whitish	Glistening, smooth	>16	>64	>128	1	>2	>128	>16	>8	>8	128	>2	>2	8	≤0.25	>16	≤1	>128	≤1	*bla*_OXA-1_, *bla*_OXA-9,_ *bla*_OXA-48,_ *bla*_TEM-1D,_ *bla*_CTX-M-15,_ *bla*_NDM-1,_ *bla*_SHV-11_
3-B			Grey	Glistening, smooth	>16	>64	>128	2	>2	>128	>16	>8	>8	128	>2	>2	8	≤0.25	>16	≤1	>128	≤1	
4-A	Intra-operatively obtained hip tissue	Sep 22	Small colony variant		>16	>64	>128	0.25	>2	>128	>16	>8	>8	128	>2	>2	32	0.5	>16	≤1	>128	>4	*bla*_OXA-1_, *bla*_OXA-9,_ *bla*_OXA-48,_ *bla*_TEM-1D,_ *bla*_NDM-1,_ *bla*_SHV-11_
4-B			Whitish	Glistening, smooth	>16	64	>128	0.125	2	≤1	≤1	≤1	8	16	>2	>2	8	0.5	>16	≤1	>128	2	*bla*_OXA-1_, *bla*_OXA-9,_ *bla*_OXA-48,_ *bla*_TEM-1D,_ *bla*_CTX-M-15_^c^ *bla*_NDM-1,_^c^ *bla*_SHV-11_
4-C			Grey	Glistening, smooth	>16	>64	>128	1	>2	>128	>16	>8	>8	128	>2	>2	8	0.5	>16	≤1	>128	4	
4-D			Grey	Dry, rough	>16	>64	>128	2	>2	>128	>16	>8	>8	64	>2	>2	8	0.5	>16	≤1	>128	≤1	
5-A	Intra-operatively obtained hip tissue	Sep 22	Small colony variant		>16	>64	>128	0.25	>2	>128	>16	>8	>8	>128	>2	>2	32	0.5	>16	≤1	>128	>4	*bla*_OXA-1_, *bla*_OXA-9,_ *bla*_OXA-48,_ *bla*_TEM-1D,_ *bla*_CTX-M-15,_ *bla*_NDM-1,_ *bla*_SHV-11_
5-B			Whitish	Glistening, smooth	>16	>64	>128	2	>2	>128	>16	>8	>8	128	>2	>2	8	0.5	>16	≤1	>128	4	
5-C			Grey	Glistening, smooth	>16	>64	>128	2	>2	>128	>16	>8	>8	128	>2	>2	8	0.5	>16	≤1	>128	4	
5-D			Grey	Dry, rough	>16	>64	>128	1	>2	>128	>16	>8	>8	64	>2	>2	8	0.5	>16	≤1	>128	4	

^a^Abbreviations of antibacterial agents; PIP, piperacillin; TZP, piperacillin-tazobactam; TEM, temocillin; FDC, cefiderocol; CTX, cefotaxime; CAZ, ceftazidime; CZA, ceftazidime-avibactam; C/T, ceftolozane-tazobactam; IPM, imipenem; MEM, meropenem; CIP, ciprofloxacin; LVX, levofloxacin; AMK, amikacin; TGC, tigecycline; CHL, chloramphenicol; CST, colistin; FOF, fosfomycin; SXT, trimethoprim-sulfamethoxazole.

^b^Cells with red background, MIC test result categorized as resistant; cells with green background, MIC test result categorized as susceptible (according to EUCAST Breakpoint tables for interpretation of MICs and zone diameters. Version 13.1, 2023);

^c^In isolate 4-B, bla_CTX-M-15_ and bla_NDM-1_ genes were detected by WGS, but not by LAMP after sub-cultivation for antimicrobial susceptibility testing.

### 2.5 DNA isolation and sequencing

After overnight growth on blood agar plates at 37 °C, ten colonies were randomly selected and suspended in 1.5 mL tubes (Carl Roth, Karlsruhe, Germany) with 1 mL of phosphate buffered saline. Total DNA was extracted using the MasterPure DNA Purification kit for Blood, v. 2 (Lucigen, Middleton, WI, USA) according to the manufacturer’s instructions. Quantification of isolated DNA was performed with the Qubit 4 fluorometer and the dsDNA HS Assay kit (Thermo Fisher Scientific, Waltham, MA, USA). DNA was sent to SeqCenter (Pittsburgh, PA, USA), where sample library preparation using the Illumina DNA Prep kit and IDT 10bp UDI indices was performed. Subsequently, libraries were sequenced on an Illumina NextSeq 2000, producing 2x151bp reads. Demultiplexing, quality control and adapter trimming at the sequencing center was performed with bcl-convert v. 3.9.3 (https://support-docs.illumina.com/SW/BCL_Convert/Content/SW/FrontPages/BCL_Convert.htm).

### 2.6 Assembly and genomic characterization

We employed a custom assembly and polishing pipeline to assemble raw sequencing reads to contigs. This pipeline consists of four parts, namely trimming (BBDuk from BBTools v. 38.98 [https://sourceforge.net/projects/bbmap/], quality control (FastQC v. 0.11.9 [https://www.bioinformatics.babraham.ac.uk/projects/fastqc/]), assembly (shovill v. 1.1.0 [https://github.com/tseemann/shovill]) with SPAdes v. 3.15.5 (Prjibelski et al., 2020), and polishing (BWA-MEM2 v. 2.2.1 (Vasimuddin et al., 2019), Polypolish v. 0.5.0 (Wick and Holt, 2022)). Genotyping was performed with Kleborate v. 2.2.0 (Lam et al., 2021) and Kaptive (Wyres et al., 2016; Lam et al., 2022).

### 2.7 Confirmation of clonality

Trimmed sequencing reads of all isolates were mapped against isolate 1-A with snippy v. 4.6.0 (https://github.com/tseemann/snippy) and the SNP distance matrix calculated with snp-dists v. 0.8.2 (https://github.com/tseemann/snp-dists).

## 3 Results

**Figure 1:**
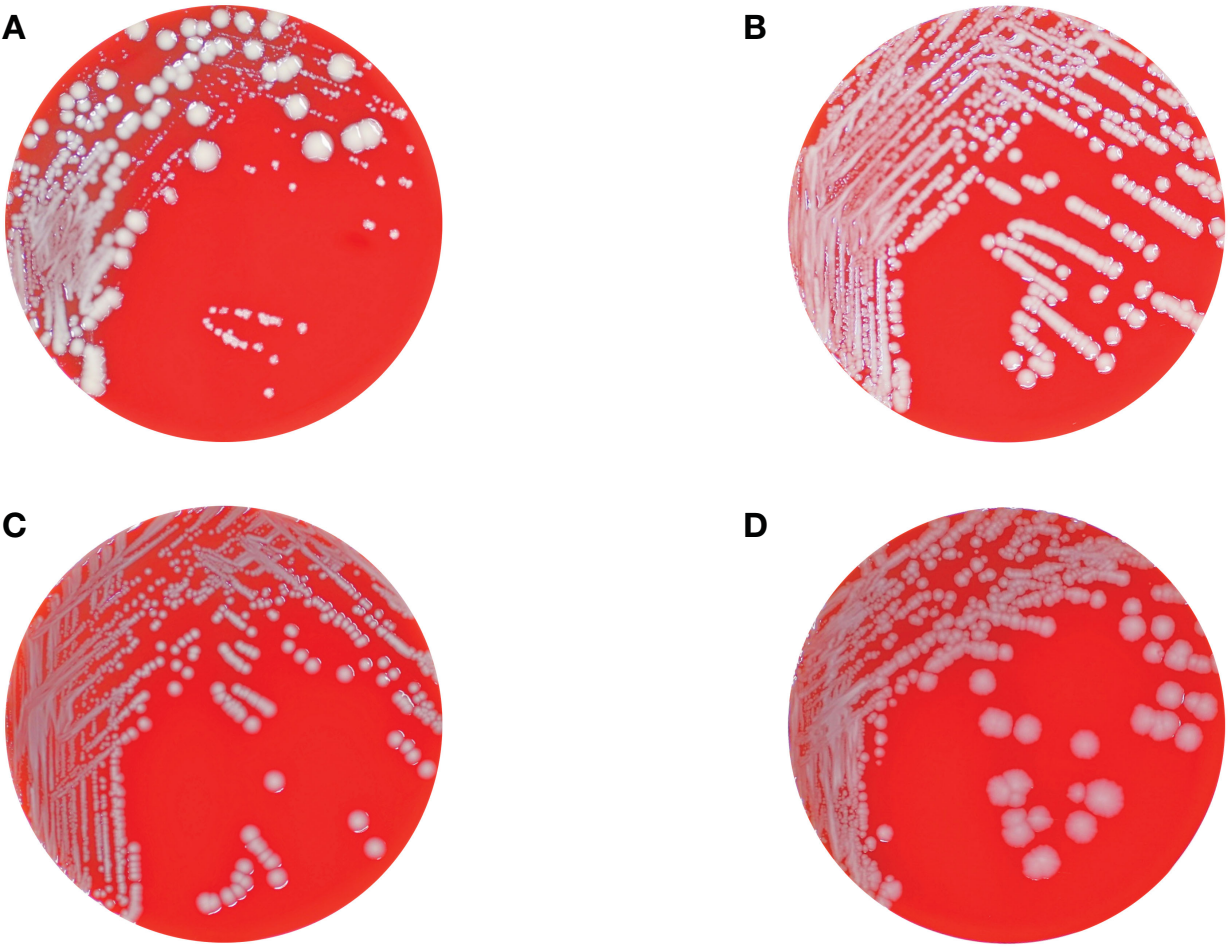
Columbia blood agar plates showing the different colonial morphotypes of the K. pneumoniae isolates comprising regular sized colonies (wild-type) with glistening whitish **(B)** and grey **(C)**, and dry and rough grey colonies **(D)**, respectively, as well as tiny grey and whitish colonies displaying the SCV phenotype **(A)**. Panel **(A)** also shows the hyper-splitting phenomenon of the SCV phenotype into the colony morphotypes shown in panels **(B–D)**.

Overall, 14 distinct phenotypes were determined (**Table 1**). From the urine, two phenotypes (1-A and 1-B) exhibiting a normal colony size and glistening surface but differing in the color of their colonies displaying whitish or grey colonies, were isolated. All other phenotypes (n = 12) were isolated from tissue specimens. Strains numbered 1-A, 2-A, 3-A, 4-B, 5-B, numbered 1-B, 2-B, 3-B, 4-C, 5-C, and numbered 4-D, 5-D, displayed identical morphological attributes each, distinguished by whitish, glistening, and smooth (**Figure 1B**), grey, glistening, and smooth (**Figure 1C**), and grey, dry, and rough colonies (**Figure 1D**), respectively (**Supplementary Figure S1**). These strains revealed a normal colony size of 2.4 mm on average (range, 1 – 5.5 mm). The isolates displaying the SCV phenotype, numbered 4-A and 5-A, exhibited similar morphological characteristics, and colony sizes were smaller than 0.5 mm (**Figure 1**, **Supplementary Figure S1**). No discernible variation in terms of colony clustering was observed among the various agar plates. There were no obvious differences in size or shape of cells between different phenotypes except bacteria from grey, dry, rough phenotype 5-D, in which cells were clearly elongated (**Supplementary Figure S2**).

Initially, largely antibiotic-susceptible *K. pneumoniae* phenotypes exhibiting whitish and grey colony morphologies on Columbia agar plates were isolated from the urine sample. Following antibiotic treatment, MDR *K. pneumoniae* strains displaying the normal colony size were isolated from tissue samples, again characterized by subsequent whitish or grey colony formations. Subsequently, SCVs of *K. pneumoniae* were isolated from tissue samples. Subcultivation of different SCV colonies consistently yielded a division into four distinct colony morphotypes including one SCV phenotype that resembled the initial SCV, along with three normal-sized phenotypes distinguished by variations in colony color and visual attributes. While normal-sized phenotypes exhibited stability following each round of re-cultivation, SCV isolates displayed instability and recurrently diverged into the four phenotypes described above. We have designated the emergence of these multiple phenotypes as “hyper-splitting”. Despite minor variations in MIC values, these “hyper-splitting” phenotypes exhibited multidrug resistance (**Table 1**).

Except for isolates 1-A and 1-B, all isolates were resistant to the tested carbapenems. Initially, during routine diagnosis, isolate 2-B was found to be carbapenem-resistant by VITEK^®^ 2 AST, and to harbor *bla*_OXA-48_ gene by LAMP. After subcultivation of this isolate for MIC determination, this resistance disappeared and the isolate became susceptible to all tested beta-lactam antibiotics except piperacillin. We assume that a mobile genetic element harboring *bla*_OXA-48_ gene was lost upon subcultivation. Only isolates 1-A and 1-B were susceptible to piperacillin, and only isolate 4-B was not resistant to the cephalosporins tested. Interestingly, only isolates 4-A and 5-A, which demonstrated the SCV phenotype, were resistant to amikacin and trimethoprim-sulfamethoxazole. Another remarkable finding was the observed increase in the MIC values of cefiderocol and trimethoprim-sulfamethoxazole over time (**Table 1**).

Whole-genome sequence (WGS) analysis revealed that all isolates belonged to sequence type (ST) 147. Lipopolysaccharide antigen (O) loci were O1/O2v1 and capsule biosynthesis (KL) loci were KL64 for all isolates except isolate 4-D, which could not be assigned, as it missed most genes of this locus. Isolates 1-A, 1-B and 2-B showed lower Kleborate resistance score than the other isolates (resistance: 0 vs. 2). The resistance score of 0 indicates that the isolate(s) did not carry any genes for extended-spectrum beta-lactamases (ESBL) or carbapenemases and a score of 2 correlated with the presence of carbapenemase genes without colistin resistance genes (Lam et al., 2021). In accordance with the resistance scores, we detected several beta-lactamase genes, such as *bla*_SHV-11_, *bla*_TEM-1_ and *bla*_OXA-9_, ESBL genes, such as *bla*_CTX-M-15_ and *bla*_OXA-1_, and the carbapenemase genes *bla*_NDM-1_ and *bla*_OXA-48_. *bla*_SHV-11_ was found in all isolates whereas *bla*_TEM-1_ and *bla*_OXA-9_ were present in all isolates except 1-A and 1-B. However, *bla*_CTX-M-15_ was not found in isolate 4-A. In isolate 4-B, *bla*_CTX-M-15_ and *bla*_NDM-1_ genes were initially detected by WGS, however, after sub-cultivation, a discrepancy between AST and WGS results was observed. Re-testing by LAMP at this later time point revealed the loss of both genes (**Table 1**). Genes associated with sulphonamide (*sul1*) and chloramphenicol (*catB3*) resistance were also detected in all isolates except 1-A, 1-B and 2-B. Note that we did not detect any common cefiderocol resistance genes.

The isolates exhibited clonality as emphasized by the low number of SNPs among them (**Supplementary Tables S1**, **S2**). Especially isolates from the same time point showed no difference in the core genome alignment (5,360,988 bp) with the exception of 2-A and 2-B (six SNPs) and 5-D (one additional SNP compared to 5-A–C). The largest distance with 17 SNPs was between 2-A and 5-D (**Supplementary Table S1**).

## 4 Discussion

When evaluating the results, we can roughly identify three distinct outcomes. The first significant observation concerns the emergence of resistance development chronologically within a *K. pneumoniae* strain, originating from a patient subjected to continuous, uninterrupted antibiotic intervention. This scenario promptly elicits contemplation of the subject concerning within-host adaptive evolution of bacteria. In fact, in-host resistance evolution, either due to plasmid mediation or chromosome mutations, has been observed even shortly after the initiation of antimicrobial treatment (Jin et al., 2021).

The second notable observation in our study is the occurrence of SCVs from patient specimens following the detection of normal-sized morphotypes. SCVs demonstrate remarkable abilities to invade and persist within host cells, thus evading the surveillance mechanisms of the immune system (Tuchscherr et al., 2020). The existence of SCVs, mostly observed in *Staphylococcus* spp., has been documented since the onset of the 20th century and has gained increasing attention due to its potential implications for both clinical and basic research (Jacobsen, 1910; Proctor et al., 2006). Regarding the SCVs of Gram-negative bacteria, studies have particularly focused on *Burkholderia* and *Pseudomonas* spp. isolated from CF patients (Oliver et al., 2000; Haussler et al., 2003a, Haussler et al., 2003b). However, there are only sparse data on the occurrence of SCV in *Klebsiella* spp (Benner et al., 1965; Musher et al., 1979; Murray and Moellering, 1982; Srinivasan et al., 2012; Silva et al., 2016).

Basically, SCVs have been determined as a subpopulation characterized by their distinct phenotypic properties, such as atypical colony morphologies including the reduced colony size (Proctor et al., 1995). Their decreased growth rate is thought to contribute to their inherent resistance, given that the decelerated growth dynamics potentially hinder the effectiveness of antibiotics geared towards rapidly proliferating cell populations (Proctor and von Humboldt, 1998). Furthermore, this phenomenon concurrently signifies decreased metabolic activity, which may engender modifications in cell wall permeability, drug uptake, or the modulation of efflux pump expression (Mitsuyama et al., 1997).

For electron transport chain-defective staphylococcal SCVs, lower efficacy of aminoglycosides known to be taken up through electrical potential across the cytoplasmic membrane (ΔΨ) was demonstrated, which is attributable to low ΔΨ (Baumert et al., 2002). These alterations could collectively contribute to enhancing resistance patterns. In this study, we observed an increase in the MIC values of amikacin, cefiderocol, and trimethoprim-sulfamethoxazole in the isolates recovered over time. This MIC increase was especially pronounced for amikacin in SCV phenotypes. Moreover, most antibiotics penetrate into host cells poorly, so the concentrations required to kill intracellularly persistent SCVs cannot be achieved (Proctor et al., 2006).

SCVs, known for their inducible formation through *in vitro* processes involving various agents, including antibiotics (Benner et al., 1965), have exhibited a propensity for increased persistence and adaptability when confronted with challenging environments (Li et al., 2016). An enhanced ability to form biofilms on biotic and abiotic surfaces has been shown for SCVs of different bacterial species (Haussler et al., 2003b; Webb et al., 2004; Al Laham et al., 2007; Allegrucci and Sauer, 2007; Millette et al., 2023). The substantial implication of SCVs extends to their involvement in biofilm development, as biofilms effectively shield bacteria from harsh host environments, thereby complicating the elucidation of drug resistance mechanisms within biofilm structures (Craft et al., 2019). Biofilms not only confer protection against host immune defenses but also serve as reservoirs for persistent infections and recurrent episodes (Mirani et al., 2015). The impact of SCV phenotype on biofilm formation in in *Klebsiella* remains to be elucidated in further studies.

Furthermore, the emergence of SCVs could plausibly be due to selection pressure from antibiotic regimens or other host-associated factors, e.g., host cationic peptides. Consistent with the case that was the subject of our study, the higher frequency of SCVs in isolates from chronic and recurrent infections compared to acute infections suggests a potential role for these variants in evading host immune responses and antimicrobial treatments (Proctor et al., 2006). In the context of our study, the emergence of SCVs after the initiation of cefiderocol treatment while already undergoing antibiotic therapy could be construed as a form of *in vivo* or *in host* induction.

The third noteworthy finding from our study underscores the inherent instability of SCVs. This dynamic interplay between stable and unstable SCVs is still poorly understood and its elucidation may contribute to a deeper understanding of their role in infection in general and persistence phenomena in particular (Becker et al., 2006). Despite comprehensive explorations largely focusing on staphylococci, a lack of investigations concerning *Klebsiella* spp. persists, and requires attention.

The observed instability among SCVs, combined with distinct antibiotic susceptibility profiles across phenotypes, increases the significance of investigating SCV plasticity (Proctor et al., 1995). Stable SCVs represent a long-term adaptation strategy, whereas their unstable counterparts may arise as stress-induced variants that result from rapid adaptation to fluctuating environments (Tuchscherr et al., 2010, Tuchscherr et al., 2011, Tuchscherr et al., 2015). This inherent instability potentially serves as a mechanism for evading host immune responses and circumventing antibiotic interventions (Tuchscherr et al., 2015). Furthermore, the involvement of epigenetic modifications, including alterations in DNA methylation patterns, could significantly influence SCV stability (Guerillot et al., 2019). In addition, regulatory systems, such as two-component systems and quorum sensing, play a crucial role in SCV formation by modulating bacterial behavior and adaptation. Disruption or dysregulation of these systems could lead to the emergence of SCVs with altered phenotypic properties (Pader et al., 2014). Due to instability, slow-growing SCVs may generate mutants that exhibit a faster growth rate than usual (Brandis et al., 2017). In instances of reversion to the wild type, rapidly growing mutant revertants may demonstrate either the loss or preservation of antibiotic resistance (Brandis et al., 2017).

A high mutation rate might favor the emergence of SCVs (Schaaff et al., 2003) and also explain the emergence of antibiotic resistance as a result of antibiotic selective pressure and the adaptation of hypermutable strains in patients, especially CF patients (Prunier et al., 2003). CF-like chronic infections have been shown to specifically contribute to the development of bacterial mutations (Smith et al., 2006). Hypermutation could result in a subpopulation of bacteria that temporarily does not grow, thus leading to persistence (Witzany et al., 2022). Additionally, an increase in the prevalence of mutator bacterial strains with deficient DNA mismatch repair (MMR) system has been detected in CF patients, who are used as a reservoir for mutation (Mena et al., 2008). To our best knowledge, we were unable to identify any instance in the available literature wherein a solitary SCV colony has given rise to four distinct colonies exhibiting disparate morphologies. Accordingly, we suggest the designation “phenotypic hyper-splitting” for this distinctive phenomenon.

We described in this study unprecedented phenotypic attributes and primarily focused on *in vitro* experiments. Therefore, the clinical relevance of our findings necessitates validation through animal models and clinical sample analyses. In this context, macrophage and neutrophil assays would be valuable for assessing both the extent of immune response and the presence of persistent cells. Moreover, the determination of the auxotrophism (Kriegeskorte et al., 2014; Becker, 2023) of *K. pneumoniae* SCVs and of the molecular mechanisms that drive SCV formation and the resulting antibiotic resistance in this species require further investigation. Integrating a comprehensive range of approaches encompassing genomics, transcriptomics, and proteomics, the utilization of experimental evolutionary models can yield valuable insights into the genetic determinants and regulatory networks orchestrating SCV phenotypes.

The genomic analysis conducted in this study has revealed clonality among all 14 isolates. Further exploration is warranted to uncover the intricate molecular mechanisms underlying phenotypic hyper-splitting and to elucidate the potential pathogenic implications of this phenomenon. To better understand the formation of the SCV phenotype especially in Gram-negative pathogens, efforts need to be intensified (i) to improve the detection and characterization of SCVs recovered from clinical samples and (ii) to elucidate their clinical impact.

## Data availability statement

The datasets presented in this study can be found in online repositories. The names of the repository/repositories and accession number(s) can be found below: https://www.ebi.ac.uk/ena, PRJEB71325.

## Ethics statement

Ethical approval was not required for the study involving humans in accordance with the local legislation and institutional requirements. Written informed consent to participate in this study was not required from the participants or the participants’ legal guardians/next of kin in accordance with the national legislation and the institutional requirements.

## Author contributions

ED: Writing – original draft, Writing – review & editing, Conceptualization, Data curation, Formal analysis, Investigation, Methodology, Project administration, Software, Visualization. KSy: Data curation, Formal analysis, Investigation, Methodology, Software, Writing – review & editing. SH: Data curation, Formal analysis, Software, Visualization, Writing – review & editing. EE: Writing – review & editing, Investigation, Data curation. GW: Validation, Writing – review & editing. RP: Validation, Writing – review & editing. JB: Validation, Writing – review & editing. EI: Writing – review & editing, Validation, Project administration, Methodology, Conceptualization. KSc: Conceptualization, Data curation, Funding acquisition, Investigation, Methodology, Project administration, Resources, Supervision, Validation, Writing – review & editing. KB: Conceptualization, Data curation, Funding acquisition, Investigation, Methodology, Project administration, Resources, Supervision, Validation, Writing – review & editing.
