# Supplementary material for: Klebsiella pneumoniae exhibiting a phenotypic hyper-splitting phenomenon including the formation of small colony variants
Source: Front Cell Infect Microbiol. 2024 Mar 27;14:1372704. doi: 10.3389/fcimb.2024.1372704 (PMC11004228; doi:10.3389/fcimb.2024.1372704)
Supplement: Supplementary file 1 [file DataSheet_1.docx]

**Supplemental material**

| **Table S1.** Core SNP distance matrix. The complete core genome alignment (gaps and ambiguous bases removed) contained 5,360,988 bp. The reference sequence for alignment was 1-A. | | | | | | | | | | | | | | |
| --- | --- | --- | --- | --- | --- | --- | --- | --- | --- | --- | --- | --- | --- | --- |
|  | **1-A** | **1-B** | **2-A** | **2-B** | **3-A** | **3-B** | **4-A** | **4-B** | **4-C** | **4-D** | **5-A** | **5-B** | **5-C** | **5-D** |
| **1-A** | 0 | 0 | 7 | 3 | 3 | 3 | 13 | 13 | 13 | 13 | 13 | 13 | 13 | 14 |
| **1-B** | 0 | 0 | 7 | 3 | 3 | 3 | 13 | 13 | 13 | 13 | 13 | 13 | 13 | 14 |
| **2-A** | 7 | 7 | 0 | 6 | 6 | 6 | 16 | 16 | 16 | 16 | 16 | 16 | 16 | 17 |
| **2-B** | 3 | 3 | 6 | 0 | 2 | 2 | 12 | 12 | 12 | 12 | 12 | 12 | 12 | 13 |
| **3-A** | 3 | 3 | 6 | 2 | 0 | 0 | 12 | 12 | 12 | 12 | 12 | 12 | 12 | 13 |
| **3-B** | 3 | 3 | 6 | 2 | 0 | 0 | 12 | 12 | 12 | 12 | 12 | 12 | 12 | 13 |
| **4-A** | 13 | 13 | 16 | 12 | 12 | 12 | 0 | 0 | 0 | 0 | 0 | 0 | 0 | 1 |
| **4-B** | 13 | 13 | 16 | 12 | 12 | 12 | 0 | 0 | 0 | 0 | 0 | 0 | 0 | 1 |
| **4-C** | 13 | 13 | 16 | 12 | 12 | 12 | 0 | 0 | 0 | 0 | 0 | 0 | 0 | 1 |
| **4-D** | 13 | 13 | 16 | 12 | 12 | 12 | 0 | 0 | 0 | 0 | 0 | 0 | 0 | 1 |
| **5-A** | 13 | 13 | 16 | 12 | 12 | 12 | 0 | 0 | 0 | 0 | 0 | 0 | 0 | 1 |
| **5-B** | 13 | 13 | 16 | 12 | 12 | 12 | 0 | 0 | 0 | 0 | 0 | 0 | 0 | 1 |
| **5-C** | 13 | 13 | 16 | 12 | 12 | 12 | 0 | 0 | 0 | 0 | 0 | 0 | 0 | 1 |
| **5-D** | 14 | 14 | 17 | 13 | 13 | 13 | 1 | 1 | 1 | 1 | 1 | 1 | 1 | 0 |

| **Table S2.** Detailed list of SNPs. The reference sequence for alignment was 1-A. ^a^ This SNP was not considered as core SNP (see Table S1) since the alignment position was not covered in all strains. CDS – coding sequence, *NA* – not applicable. | | | | | | | | | | |
| --- | --- | --- | --- | --- | --- | --- | --- | --- | --- | --- |
| **Contig** | **Position** | **Reference base** | **Alternate base** | **Type** | **Strand** | **Nucleotide position** | **Amino acid position** | **Effect** | **Product** | **Present in strain** |
| 3 | 61,264 | A | T | CDS | + | 170/1029 | 57/342 | Missense variant  c.170A>T  p.Gln57Leu | 2-oxobutyrate oxidase | 4-A 4-B 4-C 4-D 5-A 5-B 5-C 5-D |
| 3 | 163,844 | G | T | CDS | + | 294/618 | 98/205 | Missense variant  c.294G>T  p.Gln98His | Malonate decarboxylase holo-ACP synthase | 4-A 4-B 4-C 4-D 5-A 5-B 5-C 5-D |
| 3 | 246,009 | A | C | CDS | + | 1387/1653 | 463/550 | Missense variant  c.1387A>C  p.Thr463Pro | Transporter, major facilitator family | 2-A |
| 4 | 20,715 | G | A | CDS | + | 699/1584 | 233/527 | Synonymous variant  c.699G>A  p.Leu233Leu | MFS transporter | 3-A 3-B |
| 4 | 329,539 | T | G | CDS | + | 224/822 | 75/273 | Missense variant  c.224T>G  p.Ile75Ser | Cobalamin/Fe(3+)-siderophore ABC transporter ATP-binding protein | 2-B |
| 5 | 80,845 | C | T | CDS | + | 349/429 | 117/142 | Synonymous variant  c.349C>T  p.Leu117Leu | DUF805 domain-containing protein | 4-A 4-B 4-C 4-D 5-A 5-B 5-C 5-D |
| 5 | 169,993 | G | T | CDS | - | 6/213 | 2/70 | Synonymous variant  c.6C>A  p.Ser2Ser | Major cold shock protein | 5-D |
| 6 | 139,543 | C | T | CDS | - | 734/2106 | 245/701 | Stop gained  c.734G>A  p.Trp245* | Pyruvate/proton symporter CstA | 4-A 4-B 4-C 4-D 5-A 5-B 5-C 5-D |
| 6 | 139,583 | C | A | CDS | - | 694/2106 | 232/701 | Missense variant  c.694G>T  p.Ala232Ser | Pyruvate/proton symporter CstA | 4-A 4-B 4-C 4-D 5-A 5-B 5-C 5-D |
| 8 | 878 | G | A | intergenic | *NA* | *NA* | *NA* | *NA* | *NA* | 4-A 4-B 4-C 4-D 5-A 5-B 5-C 5-D |
| 10 | 83,913 | C | A | intergenic | *NA* | *NA* | *NA* | *NA* | *NA* | 4-A 4-B 4-C 4-D 5-A 5-B 5-C 5-D |
| 10 | 161,338 | C | T | CDS | + | 1702/2208 | 568/735 | Stop gained  c.1702C>T  p.Gln568* | Ferrichrome porin FhuA | 4-A 4-B 4-C 4-D 5-A 5-B 5-C 5-D |
| 12 | 46,155 | G | T | CDS | + | 1131/1437 | 377/478 | Synonymous variant  c.1131G>T  p.Leu377Leu | MFS transporter | 4-A 4-B 4-C 4-D 5-A 5-B 5-C 5-D |
| 14 | 25,555 | T | A | CDS | - | 377/489 | 126/162 | Missense variant  c.377A>T  p.Gln126Leu | PTS IIA-like nitrogen regulatory protein PtsN | 2-A 2-B 3-A 3-B 4-A 4-B 4-C 4-D 5-A 5-B 5-C 5-D |
| 14 | 97,339 | G | T | CDS | - | 578/1302 | 193/433 | Stop gained  c.578C>A  p.Ser193* | MFS transporter | 2-A 2-B 3-A 3-B 4-A 4-B 4-C 4-D 5-A 5-B 5-C 5-D |
| 14 | 101,473 | T | G | CDS | + | 29/555 | 10/184 | Missense variant  c.29T>G  p.Val10Gly | Bacterial inner membrane protein | 2-A |
| 15 | 10,164 | G | T | CDS | - | 437/561 | 146/186 | Missense variant  c.437C>A  p.Ala146Glu | Carboxymuconolactone decarboxylase family protein | 4-A 4-B 4-C 4-D 5-A 5-B 5-C 5-D |
| 19 | 88,386 | G | T | CDS | + | 966/1167 | 322/388 | Missense variant  c.966G>T  p.Gln322His | Hypothetical protein | 2-A |
| 23 | 33,304 | G | A | CDS | - | 626/951 | 209/316 | Missense variant  c.626C>T  p.Pro209Leu | Magnesium/cobalt transporter CorA | 2-A |
| 31^a^ | 5,540^a^ | A | C | CDS | + | 1615/2175 | 539/724 | Missense variant  c.1615A>C  p.Thr539Pro | Tyrosine autokinase | 2-A 2-B 3-A 3-B 4-A 4-B 4-C 5-A 5-B |
| 32 | 7,225 | A | C | CDS | + | 1003/1020 | 335/339 | Missense variant  c.1003A>C  p.Thr335Pro | Asparaginase | 2-A |
| 33 | 19,426 | C | T | CDS | - | 60/357 | 20/118 | Synonymous variant  c.60G>A  p.Leu20Leu | DUF488 domain-containing protein | 4-A 4-B 4-C 4-D 5-A 5-B 5-C 5-D |


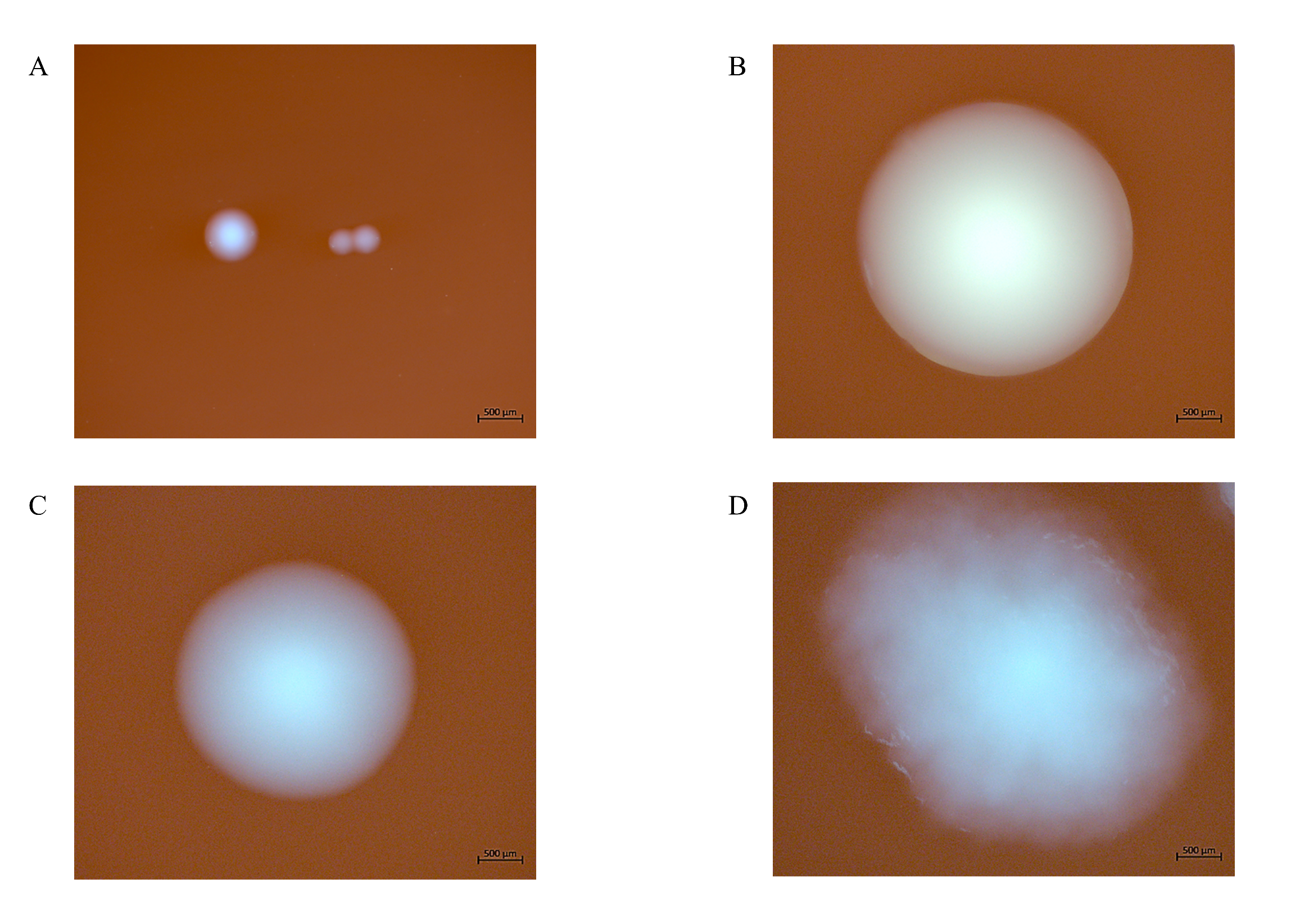


**Figure S1.** Colony morphology in different phenotypes (A: small; B: whitish, glistening, smooth; C: grey, glistening, smooth; D: grey, dry, rough colonies) using stereo zoom microscopy, 25x magnification (Zeiss Axio Zoom.V16, objective Plan Z 1.0x/0.25, Axiocam 305 camera).


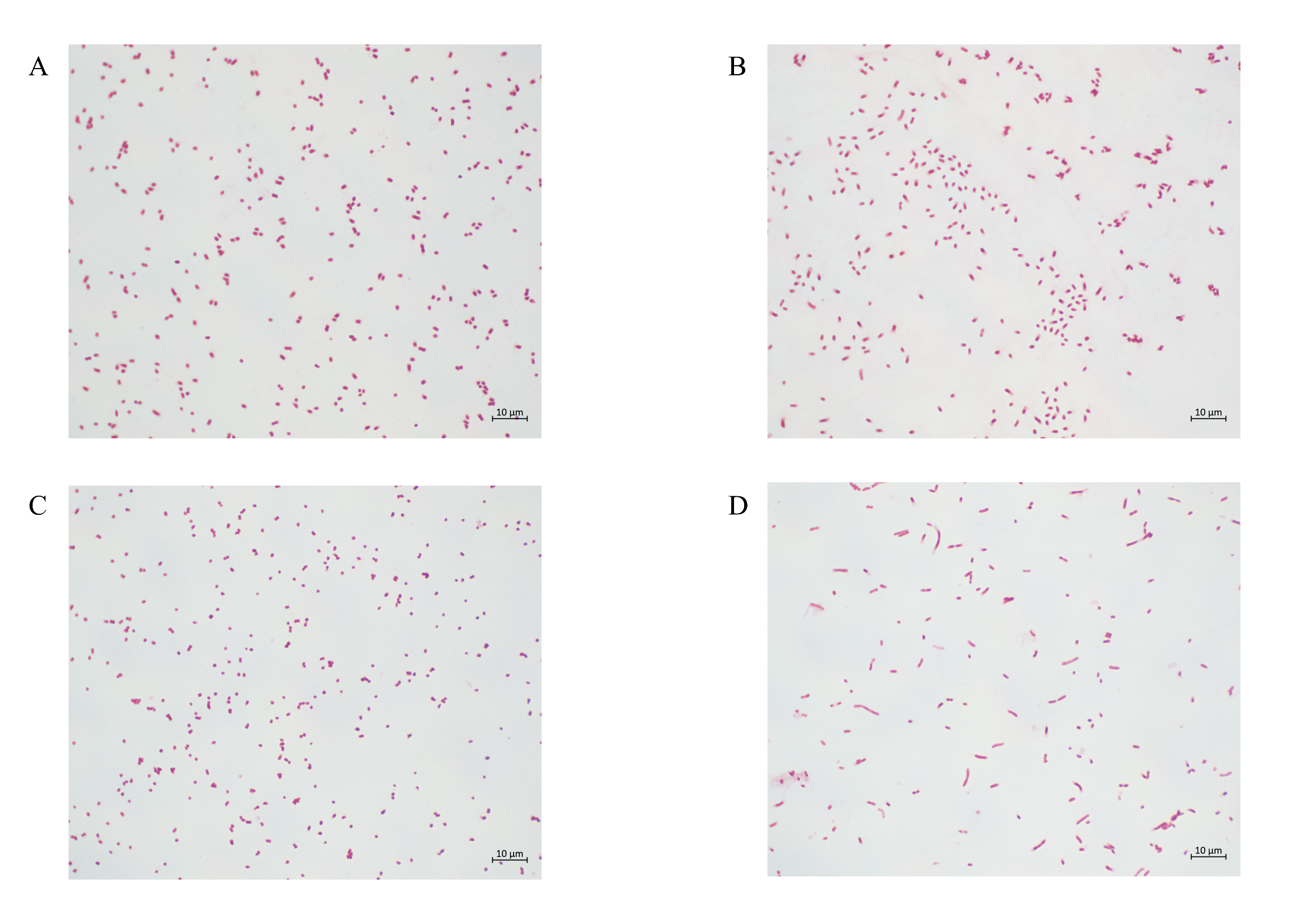


**Figure S2.** Gram staining and transmission light microscopy (Zeiss Axio Imager.Z2m, oil immersion objective Plan-APOCHROMAT 100x/1.4, Axiocam 305 camera) with different phenotypes (A: small; B: whitish, glistening, smooth; C: grey, glistening, smooth; D: grey, dry, rough colonies).
